# Prophylactic uterine artery embolization during cesarean delivery for management of hemorrhage in complete placenta previa: An observational study

**DOI:** 10.1097/MD.0000000000034052

**Published:** 2023-06-16

**Authors:** Yun He, Min Liu, Ya Jing Yang, Li Li, Qing Huang, Lanhua Liu

**Affiliations:** a Department of Obstetrics and Gynecology, Taixing People’s Hospital, TaiXing, China.

**Keywords:** complete placenta previa, hysterectomy, intraoperative blood loss, PUAE, transfusions

## Abstract

Complete placenta previa is a major cause of morbidity and mortality in pregnant women and fetuses. This study aimed to evaluate whether prophylactic uterine artery embolization (PUAE) could reduce bleeding in patients with complete placenta previa. We retrospectively analyzed patients with complete placenta previa admitted to Taixing People’s Hospital for elective cesarean delivery between January 2019 and December 2020. The women were treated with PUAE (PUAE group, n = 20) or without (control group, control, n = 20). Risk factors for bleeding (age, gestational age, pregnancy times, delivery times, cesarean delivery times), intraoperative blood loss, hemoglobin difference before and after surgery, transfusions volume, hysterectomy cases, major maternal complication cases, neonatal birth weight, neonatal Apgar score in 1 minute, postoperative hospitalization time were compared between 2 groups. There were no significant differences on risk factors for bleeding, neonatal birth weight, neonatal Apgar score in 1 minute, postoperative hospitalization time between 2 groups. However, the intraoperative blood loss, hemoglobin before and after operation, transfusion volume in the PUAE group was significantly lower than the control. There was no case of hysterectomy or major maternal complications in both groups. PUAE during cesarean may be an effective and safe strategy to reduce intraoperative blood loss and transfusion volume for patients with complete placenta previa.

## 1. Introduction

Complete Placenta previa is the placenta overlying the internal cervical OS, frequently complicated with placental accrete.^[1,2]^ It is a major risk factor for postpartum hemorrhage.^[1]^ Cesarean delivery is recommended for patients with complete placenta previa when the conditions of the fetuses are stable. However, the incidence of intraoperative and postpartum hemorrhage is still high, because bleeding cannot be avoided at the placental implantation site.^[3]^ Various conservative methods have been developed to avoid serious bleeding for complete placenta previa during cesarean section, including uterine packing,^[4]^ tourniquet method,^[5]^ uterine or iliac arteries ligation,^[6,7]^ the modified suture technique,^[8]^ balloon occlusion of the internal iliac arteries or abdominal aortic arteries,^[9,10]^ bilateral uterine arteries embolization,^[11]^ and others. Among these, it had been reported that bilateral uterine arteries embolization could significantly reduce the risk of cesarean section, hysterectomy, and transfusion for patients with placenta previa induction of labor, when the fetus is unlikely to survive.^[12–14]^ However, few data are known about whether prophylactic uterine artery embolization (PUAE) during cesarean delivery could reduce the risk of hemorrhage, transfusion, hysterectomy, and postoperative hospitalization time, in termination of pregnancy of patients with single complete placenta previa. So we aimed to evaluate the effectiveness and safety of PUAE during cesarean delivery, for controlling blood loss in patients with complete placenta previa.

## 2. Methods

### 2.1. Setting

Taixing People’s Hospital is a tertiary care referral hospital, with multidisciplinary care for the treatments of complete placenta previa. All procedures used in this study were approved by the Institute’s Ethics Committee of Taixing People’s Hospital. All participants in this study were given written informed consent. The datasets generated and (or) analyzed during the current study are available from the corresponding author on reasonable request.

### 2.2. Patients

**Figure 1.**
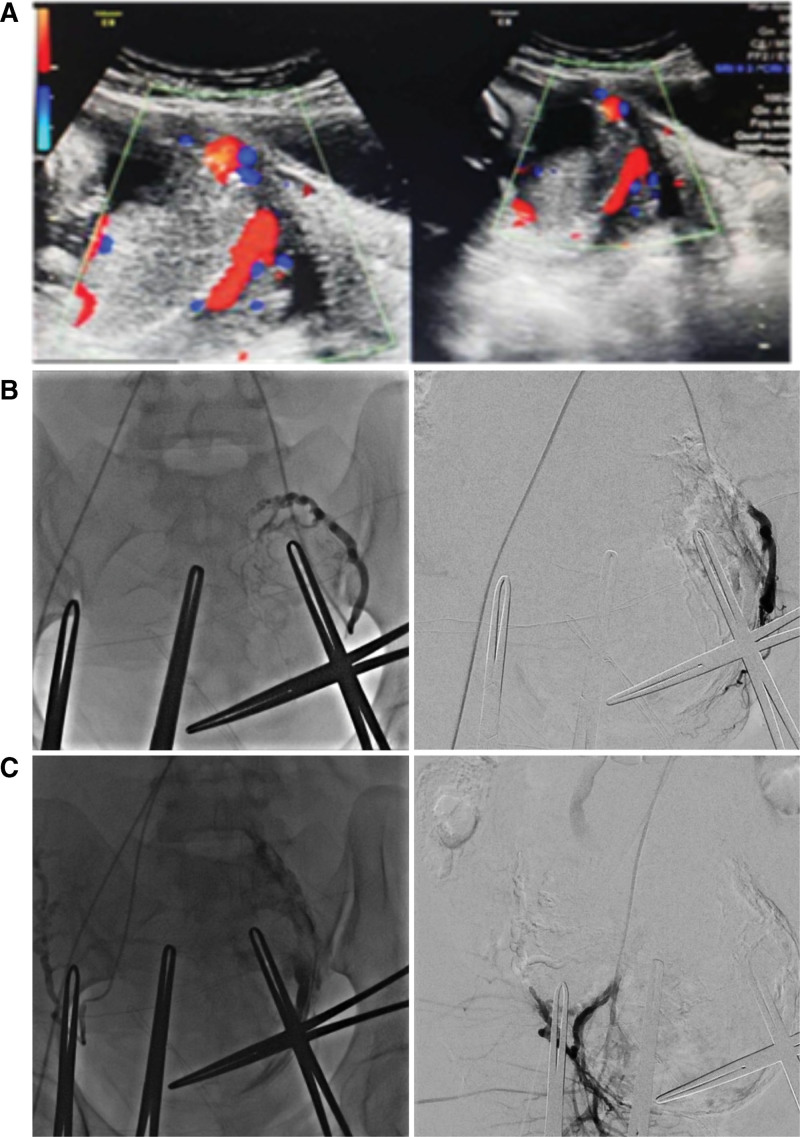
Placenta previa diagnosed by ultrasonography and effects of uterine artery embolization on the circulation of uterine arteries. (A) showed placenta completely covering cervical OS. (B) left (left uterine artery), (C) left (right uterine artery) showed the bilateral uterine arteries blood flow before uterine artery embolization. (B) right (left uterine artery), (D) right (right uterine artery) presented the bilateral uterine arteries blood flow after uterine artery embolization.

This is a retrospective analysis of patients affected by complete placenta previa in Taixing People’s hospital, from January 2019 to December 2020. An ultrasound evaluation was performed at 34 weeks of gestation to diagnose complete placenta previa. In this study we included only patients with complete placenta previa defined as: placenta previa completely covering cervical OS. All patients with complete placenta previa was evaluated by Ultrasound Scoring System before operation, and ultrasound score ≥ 6 points was identified as suspicious placenta accreta spectrum (PAS) disorder as previous published article,^[15–17]^ and patients with suspicious PAS disorder were excluded from the study. All patients in this study were well planned and prepared before operation, elective cesarean section was scheduled between 36 and 38 weeks according to Clinical Practice Guideline. Emergency cesarean section, prepartum hemorrhage, or other complications of pregnancy were also excluded. A total of 40 patients with complete placenta previa were enrolled in this study. All cases were treated by PUAE or not during cesarean surgery, the complete placenta previa was diagnosed by ultrasonography and confirmed by intraoperatively (Fig. 1A). According to whether treated with PUAE, patients were divided into control (CON) group (control group, perform only cesarean section, n = 20) and PUAE group (prophylactic uterine artery embolization group, perform prophylactic uterine artery embolization before cesarean section, n = 20).

### 2.3. Bilateral uterine arteries embolization in cesarean surgery

According to women’s choice, and the final decision to perform the PUAE at our institution was made by consensus after multidisciplinary discussion of the Obstetrics, Intervention Radiology, and Anesthesiology. Written informed consent for the PUAE procedure was obtained. Adequate blood products were prepared before surgery. All operations in this study were performed by the same surgical team, including 2 senior obstetricians with 20 years of surgical experience, 1 senior interventional radiologist, 1 senior pediatrician, 1 senior anesthesiologist, and 3 experienced nurses. When the operating field was prepared, a femoral catheter was utilized to catheterize the uterine arteries. Then the cesarean was performed. After fetal delivery, 4 Alis forceps were used to keep uterine incision from bleeding temporarily. Before placenta delivered, absorbable gelatin sponge particles were used to embolism bilateral uterine arteries. After bilateral uterine arteries were embolized, and the blood flow was significantly decreased (Figs. 1B-C), then the placenta was removed. In control group, the cesarean section was performed without bilateral uterine arteries embolization. Intraoperative blood loss was evaluated via the volume of blood aspirated from the surgical field and absorbed by gauzes. If hemoglobin value was lower than 7 g/dL, transfusion would be carried out.

### 2.4. Statistical analysis

All data expressed as mean ± SEM. Statistical analysis comparing both groups was performed using the *t* test, and *P* value < .05 was considered statistically significant. Kolmogorov-Smirnov test was used to assess data distribution. All data were analyzed by Graph Pad Prism8 software (Graph Pad, La Jolla, CA).

## 3. Results

**Figure 2.**
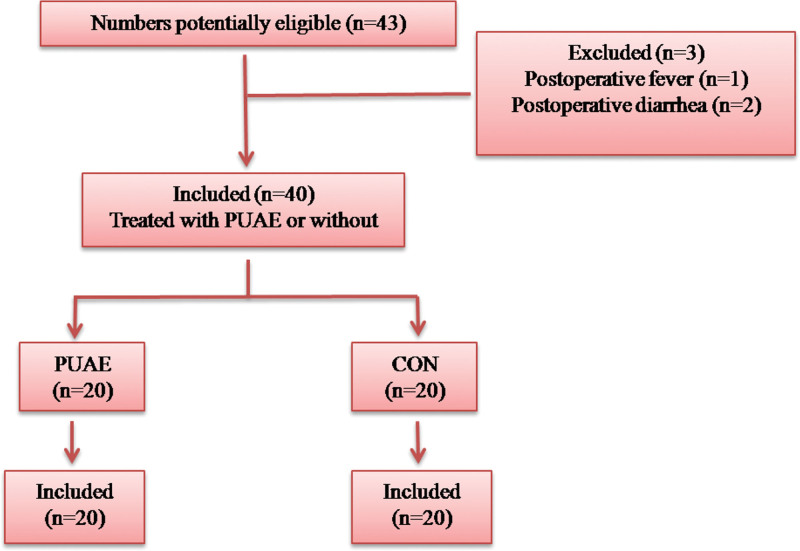
Data collected for study. A total of 40 people were included in this study after screening, exclusion and loss of follow-up. There were 20 participants in the PUAE group and 20 participants in the CON group. CON = control. PUAE = prophylactic uterine artery embolization.

A total of 40 people were included in this study after screening, exclusion and loss of follow-up. There were 20 participants in the PUAE group and 20 participants in the CON group. Data for each variable of interest were collected (Fig. 2).

### 3.1. Risk factors for bleeding in patients with complete placenta in the two groups

The study included 20 and 20 women in the CON group and the PUAE group respectively. In the CON group, the mean ± SEM age was 31.50 ± 1.21 years, the gestational age was 36.8 ± 0.4 weeks, pregnancy times was 2.90 ± 0.33, delivery times was 1.76 ± 0.14 and cesarean delivery times was 0.60 ± 0.13. In the PUAE group, the mean ± SEM age was 32.15 ± 1.28 years, the gestational age was 36.7 ± 0.3 weeks, pregnancy times was 2.45 ± 0.32, delivery times was 1.60 ± 0.11 and cesarean delivery times was 0.55 ± 0.15. There were no significant differences on age, gestational age, pregnancy times, delivery times, cesarean delivery times between the PUAE group and the CON group (Table 1).

### 3.2. Intraoperative bleeding was lower in the PUAE group

**Figure 3.**
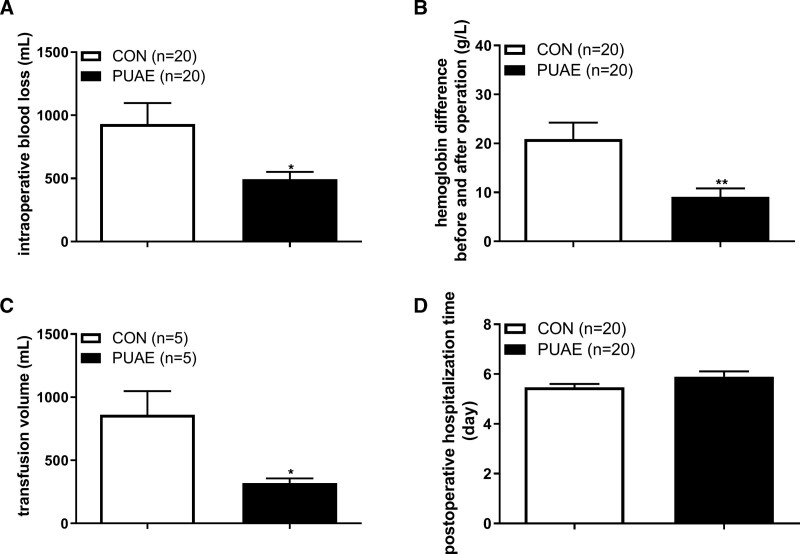
The effects of PUAE on hemorrhage volume, hemoglobin changes, transfusion volume, and post operateive hospitalization time. Figure 2A–D showed intraoperative blood loss, hemoglobin difference before and after operation, transfusion volume, and postoperative hospitalization time in both PUAE group and CON group. CON: treated without PUAE, PUAE: treated with PUAE. *, *P* < .05. **, *P* < .01. CON = control, PUAE = prophylactic uterine artery embolization.

The amount of intraoperative blood loss, hemoglobin difference before and after operation, transfusion volume in the PUAE group was (494 ± 58) mL, (9.1 ± 1.72) g/L, (320 ± 37) mL respectively, those significantly lower than that in the CON group (930 ± 166) mL, (20.9 ± 3.35) g/L, (860 ± 188) mL (Figs. 3A–C).

### 3.3. Comparison of postoperative hospitalization time between two groups

In the CON group, the mean ± SEM postoperative hospitalization time was 5.47 ± 0.13 days, in PUAE group, the mean ± SEM postoperative hospitalization time was 5.90 ± 0.21 days. There were no significant differences on postoperative hospitalization time between the PUAE group and the CON group (Fig. 3D).

### 3.4. Comparison of hysterectomy, maternal and newborn complications between two groups

There was no case of hysterectomy or major maternal complications in both groups. In the CON group, the mean ± SEM neonatal birth weight was 2965 ± 113.3g, neonatal Apgar score in 1 minute was 9.30 ± 0.333. In PUAE group, the mean ± SEM neonatal weight was 3035 ± 124.8g, neonatal Apgar score in 1 minute was 9.20 ± 0.213. There were no significant differences on neonatal birth weight, and neonatal Apgar score in 1 minute between the PUAE group and the CON group (Table 2).

## 4. Discussion

In this study, we demonstrated prophylactic uterine artery embolization during cesarean delivery for patients with complete placenta previa was associated with lower intraoperative blood loss, hemoglobin difference before and after operation, and transfusion volume. However, the postoperative hospitalization time, mother and newborn complications were not observably increased. Therefore, prophylactic uterine artery embolization during cesarean delivery was an effective and safe measure to reduce intraoperative bleeding, for patients with complete placenta previa.

Complete placenta previa is described as placenta extends completely over the internal cervical OS. Complete placenta previa often complicated by placenta accrete that can unexpectedly lead to postpartum hemorrhage, transfusion, infection, emergency hysterectomy and even death. The hemorrhage risk during cesarean delivery in the case of complete placenta previa is higher when compared to patients without it. The cause of placenta previa is unclear, might due to maternal age, abortion, previous cesarean section and history of uterine surgery.^[18,19]^ It had been reported that several risk factors contributed to postpartum hemorrhage, such as age, abortion, multiparty, previous cesarean, birth weight and so on.^[20]^ In this study, the clinical epidemiologic data showed that the risk factors for bleeding, including age, gestational age, pregnancy times, delivery times, cesarean delivery times and neonatal birth weight were not significant differences between the PUAE group and the control group. Hence, in this study, the risk factors for bleeding of 2 groups were equal.

Uterine artery embolization for the treatment of postpartum hemorrhage is widely used to avoid hysterectomy, however, prophylactic uterine arteries embolization still remains controversial and requires further evaluations.^[14,21]^ The present study used prophylactic uterine artery embolization during cesarean delivery for women with complete placenta previa, to decrease intraoperative blood loss, the results showed that prophylactic uterine artery embolization during cesarean delivery could significantly reduce intraoperative blood loss, hemoglobin difference before and after operation and transfusion volume, among patients with complete placenta previa compared to control group, indicating that the blood loss could be reduced by prophylactic uterine artery embolization during cesarean delivery. These observations suggested that prophylactic uterine artery embolization during cesarean delivery was effective at preventing intraoperative blood loss and transfusion volume in pregnant women with complete placenta previa.

Recent studies showed that application of uterine artery embolization before delivery could effective to reduce hysterectomy frequency in patients with placenta previa.^[22–24]^ In present study, there was no case of hysterectomy in both groups. However, there are still some complications of prophylactic uterine artery embolization during delivery, such as post-embolization syndrome, endometritis, peritonitis, fistula maternal and others.^[21,25]^ Hence, a growing debate on the safety of uses uterine artery embolization as a strategy to prevent severe postpartum hemorrhage.^[26]^ In our study, no such complications were occurred in both groups. Nevertheless, uterine artery embolization before delivery might be associated with fetal and neonatal complications, such as acute hypoxia, exposure to radiation.^[27,28]^ In this study, we treated patients with uterine artery embolization immediately after baby delivery, to avoid fetus exposure to radiations. In addition, the results showed that there were no significant differences on neonatal Apgar score in 1 minute between 2 groups. Our results appear to application of prophylactic uterine artery embolization during cesarean delivery, for reduce intraoperative blood loss and transfusion volume in complete placenta previa pregnant women is safety.

It had been reported that prophylactic uterine artery embolization during delivery requires longer postoperative hospitalization time and higher cost.^[14]^ In present study, there were no significant differences on postoperative hospitalization time between the PUAE group and the control group, suggesting prophylactic uterine artery embolization during cesarean delivery in complete placenta previa pregnant women did not expend longer postoperative hospitalization time.

PAS disorder is defined as trophoblastic attachment to the myometrium without intervening decidua, it is associated with maternal and fetal morbidity and mortality for the reasons of massive hemorrhage and preterm birth. Previous studies showed that prophylactic internal iliac artery balloon catheterization reduced intraoperative blood loss and transfusion, lessened perioperative hemostatic measures and reduced hysterectomies in PAS disorder.^[29,30]^ Other work suggested that prophylactic intraoperative abdominal aortic balloon occlusion could not control massive hemorrhage in patients with PAS disorder during cesarean delivery.^[31]^ However, there is limited information on the roles of prophylactic uterine artery embolization in PAS disorder. It is interesting to conduct a research on the effects of prophylactic uterine artery embolization on PAS disorder in the future studies. Since the present study did not include such research, we should not simply exclude possible role of prophylactic uterine artery embolization on the PAS disorder.

This study also had some limitations. We only observed short-term postoperative effects, mid - and long-term follow-up observations were lacking in this study. And due to the limitations of number of cases, only 40 clinical cases were included in this study, and the limited data collected may affect the results of the study. A multi-center, large sample, randomized controlled study and mid - and long-term follow-up observations was necessary in future studies.

## 5. Conclusion

Great attention has been paid to the potential role of interventional techniques in the control of peripartum hemorrhage in patients with placenta previa especially complicated with PAS or uterine scar. However, the effects of prophylactic uterine artery embolization in management of intraoperative hemorrhage in complete placenta previa without complications in the third trimester of pregnancy are little known. The present study has demonstrated that prophylactic uterine artery embolization during cesarean delivery improved maternal and fetus outcomes in patients with single complete placenta previa. These attributed to the significantly lower intraoperative blood loss and transfusion. Longer postoperative hospitalization time and major maternity and neonatal complications did not observed in this study. The data gained provided an important clue for the treatment of single complete placenta previa in the third trimester of pregnancy.

**Table 1:** Risk factors for bleeding in two groups (mean ± SEM).

	CON (n = 20)	PUAE (n = 20)	*P* value
Age (yr)	31.50 ± 1.21	32.15 ± 1.28	.714
Gestational age (wk)	36.8 ± 0.4	36.7 ± 0.3	.829
Pregnancy times	2.90 ± 0.33	2.45 ± 0.32	.335
Delivery times	1.76 ± 0.14	1.60 ± 0.11	.579
Cesarean delivery times	0.60 ± 0.13	0.55 ± 0.15	.246

PUAE = prophylactic uterine artery embolization.

**Table 2:** Maternal and neonatal complications in two groups (mean ± SEM).

	CON (n = 20)	PUAE (n = 20)	*P* value
Hysterectomy cases	0	0	
Major maternal complications	0	0	
Neonatal birth weight (g)	2965 ± 113.3	3035 ± 124.8	.678
Apgar score in one minute	9.30 ± 0.333	9.20 ± 0.213	.802

## Acknowledgments

Yun He, Lanhua Liu designed the experiments, Lanhua Liu, Yun He, MinLiu, Yajing Yang performed the surgery, LiLi and Qing Huang did the post-operation care. Yun He, Min Liu accomplished the date statistics analysis, and wrote the manuscript, Lanhua Liu revised it. This work was supported by Maternal and child health research project of Jiangsu Province (F202203), Taixing People’s Hospital Foundation (try1816), and Postgraduate Research & Practice Innovation Program of Jiangsu Province (KYCX20-2693).

## Author contributions

**Conceptualization:** Yun He, Qing Huang.

**Data curation:** Yun He, Min Liu, Ya Jing Yang, Qing Huang, Lanhua Liu.

**Formal analysis:** Yun He, Min Liu, Lanhua Liu.

**Funding acquisition:** Yun He.

**Investigation:** Yun He, Min Liu, Ya Jing Yang, Li Li, Lanhua Liu.

**Methodology:** Yun He, Min Liu, Li Li, Lanhua Liu.

**Project administration:** Yun He, Lanhua Liu.

**Supervision:** Yun He.

**Software:** Ya Jing Yang.

**Writing – original draft:** Yun He, Min Liu.

**Writing – review & editing:** Lanhua Liu.
